# Use of a Novel, Remotely Connected Diabetes Management System Is Associated with Increased Treatment Satisfaction, Reduced Diabetes Distress, and Improved Glycemic Control in Individuals with Insulin-Treated Diabetes: First Results from the Personal Diabetes Management Study

**DOI:** 10.1089/dia.2017.0206

**Published:** 2017-12-01

**Authors:** Pablo Mora, Ann Buskirk, Maureen Lyden, Christopher G. Parkin, Lena Borsa, Bettina Petersen

**Affiliations:** ^1^ Dallas Diabetes Research Center, Dallas, Texas.; ^2^ Roche Diabetes Care, Indianapolis, Indiana.; ^3^ Biostat International, Tampa, Florida.; ^4^ CGParkin Communications, Inc., Boulder City, Nevada.; ^5^ Roche Diabetes Care GmbH, Mannheim, Germany.

**Keywords:** mHealth, Type 1 diabetes, Type 2 diabetes, Insulin, Multiple daily injections, Self-monitoring of blood glucose.

## Abstract

***Background:*** The ability to automatically transfer data to clinicians and receive timely guidance in therapy adjustments through remote and in-office consults can positively impact patients' perceptions about quality of care, which is positively associated with clinical outcomes. We assessed the impact of using the Accu-Chek Connect diabetes management system on treatment satisfaction, diabetes distress, and glycemic control in adults with type 1 diabetes and insulin-treated type 2 diabetes.

***Subjects and Methods:*** This 6-month, prospective, multicenter, single-arm study assessed the impact of using the system on treatment satisfaction and glycemic control among 87 adults with insulin-treated diabetes (multiple daily insulin injections and basal only), with 8.8% ± 1.6% glycated hemoglobin (HbA1c) at baseline. The Diabetes Treatment Satisfaction Questionnaire-status (DTSQs) and Diabetes Distress Scale (DDS) were administered at baseline, and the Diabetes Treatment Satisfaction Questionnaire-change (DTSQc) and DDS at 6 months. Changes in HbA1c, average blood glucose (BG), and other metrics were also assessed.

***Results:*** Improvements in DTSQc scores were observed at 6 months with a total mean (standard deviation) score of 14.3 ± 5.1. Significant reductions in total mean DDS scores from baseline to 6 months were also observed, from 2.0 ± 0.8 to 1.7 ± 0.7, *P* < 0.0001. A significant reduction in regimen-related distress was notable, from “moderate distress” (2.4 ± 1.0) to “not distressed” (1.9 ± 0.9), *P* < 0.0001). Significant reductions in mean HbA1c (−0.9 ± 1.6, *P* < 0.0001) and mean BG (−24.8 ± 50.8, *P* < 0.0001) were observed.

***Conclusions:*** Use of the Accu-Chek Connect diabetes management system is associated with increased treatment satisfaction and improved glycemic control among individuals with insulin-treated diabetes. NCT02600845 (www.clinicaltrials.gov).

## Introduction

The routine availability and appropriate use of structured self-monitoring of blood glucose (SMBG) data facilitate earlier, more frequent treatment interventions and improve glycemic control in individuals with type 1 diabetes (T1D) and type 2 diabetes (T2D).^1–4^ However, obtaining accurate and complete glucose data is often problematic. Traditional SMBG logbooks are often erroneous and deficient, and many patients forget to bring their glucose records to clinic visits.^5–7^ It also has been well documented that many clinicians do not intensify diabetes therapies in a timely manner in patients who are above their recommended glycemic targets.^8–12^

Patients' perceptions about the quality of their care and treatment satisfaction are key contributors to optimal clinical outcomes.^13,14^ In a recent systematic review, Doyle et al. found significant associations between positive patient experience, clinical effectiveness, and safety across a range of diseases, including diabetes.^13^ It has also been shown that clinicians play a major role in promoting treatment satisfaction through good communication with their patients.^15^

Use of mobile phone interventions for diabetes self-management can significantly improve glycemic control in individuals with diabetes by promoting improvement in diabetes self-management activities.^16–18^ With the increasing number of smartphone users, which is expected to rise to 6.5 billion by 2018,^19^ this technology offers the potential to help patients and their clinicians better manage diabetes. However, the potential value of electronic diabetes management technology that automates visualization, transfer, and triage of SMBG data and other diabetes treatment information has not been well studied.

**FIG. 1.**
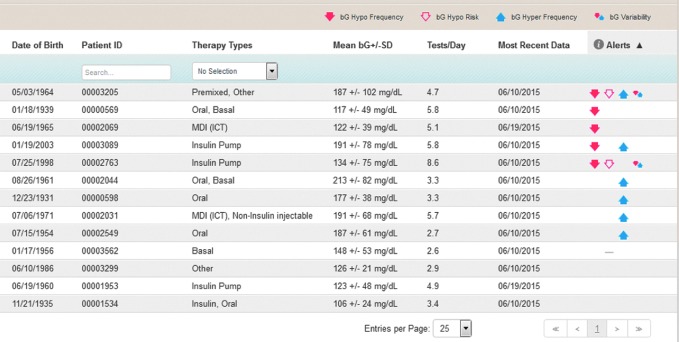
Clinician web portal triage feature. The software clusters all patients with “BG hypo frequency” as the most serious risk, followed by “BG hypo risk,” “BG hyper frequency,” and “BG variability.” Glycemic thresholds for these risk categories can be individualized by the clinician. BG, blood glucose; ICT, intensive conventional therapy; MDI, multiple daily insulin injections.

The Accu-Chek^®^ Connect diabetes management system (Roche Diabetes Care, Indianapolis, IN) presents a novel approach to digital diabetes management. The system consists of a blood glucose (BG) meter, smartphone application (app), and an online data management web portal. The meter connects wirelessly, by Bluetooth^®^ low energy technology, to the user's smartphone app, which provides multiple functions to facilitate diabetes management. The system provides automatic, wireless transfer of BG results from meter to the app and then to secure personal and clinical web portals. Users also have the option to share glucose data with others through text message. A key feature of the system is the clinician portal home page, which automatically organizes the patient data, identifying patients who are at risk for acute glycemic events, thereby providing clinicians the ability to triage patients according to greatest need. Manual download of data is unnecessary (Fig. 1).

We hypothesized that the ability to automatically transfer diabetes data to clinicians and receive timely guidance in therapy adjustments through remote consults (text or phone) and in-office consults may positively impact patients' perceptions about quality of their care and lead to improved glycemic control. To test this hypothesis, we assessed the impact of system use in individuals with T1D and insulin-treated T2D.

## Methods

### Study design

The Personal Diabetes Management (PDM) trial was a 6-month, interventional, single-arm prospective multicenter study. The primary objective of this study is to assess change in treatment satisfaction of T1D and insulin-treated T2D patients who utilize the system over a period of 6 months. Secondary outcomes included the following: change in diabetes-related distress; changes in glycemic control (glycated hemoglobin [HbA1c] and mean BG; and glycemic variability over time); change in daily SMBG frequency; and impact of structured diabetes data on clinician ability to make informed decisions on diabetes and adjust medication.

### Subjects

Patients were recruited from 12 clinical sites in the United States; sites included 6 primary care practices and 6 diabetes specialty practices. The targeted study population included adults ≥18 years of age with poorly controlled T1D and insulin-treated T2D patients who were experienced with Smartphone use (>3 months) and had downloaded at least one app. Recruitment occurred over 3 months at each site. Subjects were recruited based on their own interest to participate in this study as well as verification of their eligibility.

#### Inclusion criteria

Inclusion criteria were ≥18 years of age; diagnosis of T1D or T2D ≥6 months; currently using insulin as a component of the diabetes therapy; able to provide SMBG data up to 1 month before study start; SMBG frequency, as confirmed by download (basal insulin-treated subjects: SMBG ≥5 × /week; multiple daily insulin injection [MDI]: SMBG ≥2 × /day; ≥7.5% HbA1c (per local laboratory obtained ≤3 months of baseline)); able to read and write in English language; and currently using a Smartphone and have experience with downloading at minimum one app. Smartphone compatibility—must be able to download the system app; naive to the system; and willing to comply with study procedures.

#### Exclusion criteria

Exclusion criteria were treatment with continuous subcutaneous insulin infusion; any use of continuous glucose monitoring (CGM) to manage their diabetes during the course of the study; pregnant, lactating, or planning to become pregnant during the study period; diagnosed with any clinically significant condition (e.g., anemia, major organ system disease, infections, psychosis, or cognitive impairment); or requires chronic steroid in adrenal suppressive doses, other immunomodulatory medication, or chemotherapy.

The study protocol was approved by a local institutional review board for each investigator site and is in compliance with the Helsinki Declaration.^20^ A signed informed consent was obtained from all subjects.

### Procedures

The study's duration was 6 months with patient visits occurring at initial screening and baseline followed by visits at months 3 and 6, with a training check phone call at week 1.

#### Visit 1 (baseline)

At this clinic visit, investigators obtained signed informed consent, assessed all applicable inclusion/exclusion criteria, and documented demographic data, medical history, and current medications. Investigators then administered the patient prequestionnaire, which included the Diabetes Treatment Satisfaction Questionnaire-status (DTSQs) and Diabetes Distress Scale (DDS) to benchmark current levels of treatment satisfaction and diabetes-related distress. A physical examination was performed and laboratory samples were obtained. Glucose data from patient BG meters were downloaded and assessed to confirm patient eligibility. Investigators reviewed therapy/insulin parameters, discussed the downloaded SMBG data, and modified each patient's regimen, as needed. Patients were instructed to perform 3-day/7-point profile glucose testing or follow an individualized, structured testing regimen. Patients were provided an SMBG testing regimen instruction sheet and instructed to follow their SMBG regimen. Investigator staff trained the patient on system setup (connectivity to the smartphone app and web portals) and use. At the visit and subsequent scheduled visits, clinicians were asked to assess and document the sufficiency of patient diabetes data to make informed decisions regarding therapy changes (adjust medication, lifestyle/behavioral counseling, address skill deficit, and address nonadherence). All therapy changes were documented.

#### Visit 2 (week 1)

At this remote visit, investigators called each patient to identify any issues/obstacles associated with system use.

#### Visit 3 (month 3)

At this clinic visit, investigators obtained a finger-stick sample for central laboratory measurement of HbA1c and used a checklist to review/discuss data with patient, collaboratively agree on therapy changes (pharmacologic and/or behavioral), and modify the SMBG regimen if needed. The sufficiency of the SMBG data provided between visits 1 and 3 to make informed decisions regarding patient therapy adjustment was assessed and documented. If no treatment changes were made, patients were asked to follow the therapy recommendation and repeat 3-day/7-point profile or physician-prescribed testing regimen. If a treatment change was required, investigators prescribed the new therapy recommendation/SMBG regimen and asked the patient to follow this recommendation. Adverse events (AE) and serious adverse events (SAE) were assessed and documented.

#### Visit 4 (month 6)

At this clinic visit, investigators obtained a finger-stick sample for HbA1c measurement and used a checklist to review/discuss data with patient, collaboratively agree on therapy changes (pharmacologic and/or behavioral), and modify the SMBG regimen if needed. The sufficiency of the SMBG data provided between visits 3 and 4 was assessed and documented. Investigators then administered the patient postquestionnaire—Diabetes Treatment Satisfaction Questionnaire-change (DTSQc), DDS, and investigator-developed preference questionnaire—to assess changes in treatment satisfaction and diabetes-related distress, and to evaluate patient preference for the system compared with their previous technology and/or diabetes treatment process. AE and SAE were assessed and documented. If ongoing AE were reported at visit 4, a follow-up phone call was placed 2–5 days after visit 4.

#### Unscheduled visits

Throughout the study, clinicians were instructed to monitor the clinician web portal home page once a week to identify high-risk patients who may require an unscheduled visit to address the concern. Unscheduled visits were conducted in clinic or remote (through telephone), and therapy changes were documented.

### Outcome measures

The primary outcome measure of this study was to assess change in treatment satisfaction of patients who utilized the system over a period of 6 months, using the DTSQ to determine baseline satisfaction (DTSQs), and change in treatment satisfaction (DTSQc).^21^

Secondary measures included: change in diabetes-related distress as assessed by the DDS^22^; changes in glycemic control (HbA1c and mean BG); change in daily SMBG frequency; impact of structured diabetes data on healthcare professional's (HCP) ability to make informed decisions on diabetes management therapy changes; and number of unscheduled patient visits/consults.

### Statistical analysis

Assuming a significance level of 0.05 (two-sided) and 80% power, an improvement in satisfaction, defined as a DTSQc score of at least 2 (standard deviation [SD] = 7), can be detected with a sample size of 99 subjects. The DTSQc score obtained at visit 4 is summarized by descriptive statistics (*n*, mean, SD, median, range, and 95% confidence interval of the mean).

The primary analysis population for the study is the full analysis set (FAS), which is defined as all enrolled and trained patients who provided data using the system within 14 days of visit 4 and completed the DTSQc at visit 4. SAS Version 9.2 or higher was used for all statistical analyses.

The primary endpoint was the change in satisfaction score (DTSQc) in patients who utilized the system. The DTSQs was administered at baseline to determine patient treatment satisfaction before system use, which was calculated as the sum of the items (except items 2 and 3). The total change score (DTSQc) at the end of the study was similarly calculated. The mean DTSQc score at visit 4 was compared to the hypothesized value of zero using a one-sample *t*-test; *P*-values from the one-sample *t*-test are presented.

#### Diabetes-related distress

Diabetes-related distress was measured using the DDS, a validated17-item measure that uses a Likert scale to grade each item from 1 (no problem) to 6 (a very serious problem) scale concerning the subject's diabetes distress experienced over the last month.^22^ This scale yields four subscales that target different areas of potential diabetes-specific distress: emotional burdens (feeling overwhelmed by diabetes), physician-related distress (worries about access, trust, and care), regimen-related distress (concerns about diet, physical activities, and medication), and interpersonal distress (not receiving understanding and appropriate support from others). The total DDS score was derived as a mean score by summing the patient's responses to the 17 items and dividing by the number of items in the scale. The change from baseline in the total diabetes-related distress score at visit 4 is summarized using descriptive statistics (*n*, mean, SD, median, range, and 95% confidence interval of the mean). The mean change from baseline in the DDS score at visit 4 was compared to the hypothetical assumption of no change (*d* = 0) using a paired *t*-test; *P*-values from the paired *t*-test are presented.

#### Glycemic measures

Capillary/finger-stick blood samples were obtained for the central laboratory determination of HbA1c at visits 1, 3, and 4. The changes from baseline in the HbA1c at visit 3 (12 weeks) and visit 4 (month 6) are summarized using descriptive statistics (*n*, mean, SD, median, range, and 95% confidence interval of the mean) The mean of BG values obtained 30 days before visits 3 and 4 are summarized (*n*, mean, SD, median, and range). The mean BG change from baseline at visit 4 was measured using a paired *t*-test; *P*-values are presented.

#### SMBG frequency

The average number of daily SMBG measurements within the interval is summarized using descriptive statistics (*n*, mean, SD, median, and range) by study visits.

#### Sufficiency of diabetes data

The number and percentage of patients who provided diabetes data that was sufficient to make an informed treatment adjustment were calculated and reported as a total number and percent of subjects at each visit. The change within patients from baseline to visits 3 and 4 in data sufficiency was compared by a McNemar's test. The number and percent of subjects who were prescribed different treatment changes at each visit were also summarized.

#### Unscheduled medical visits/consults

The number and percentage of patients who had an unscheduled clinic visit and/or remote consult were calculated and reported as follows: total number of patients; total number of visits; type of visit (clinic or remote); total number initiated by clinicians and patients; and nature of visit (medication change and education/training only).

## Results

The study enrolled 122 patients at 12 sites; 87 patients met the FAS criteria and are included in this analysis. Five patients withdrew consent, 27 patients were noncompliant with study procedures, and 3 discontinued for other reasons. One clinical site was discontinued from the study due to noncompliance with the study requirements; this accounted for 10 of the 27 patients who were not included in the FAS analysis. The FAS cohort was an older population, well educated, and predominantly white. Most patients had T2D and were treated with MDI therapy (Table 1).

**Table 1.** Demographic Characteristics

*Characteristic*	n* = 87*
Age, years (SD)	57.9 (12.0)
Female, *n* (%)	45 (51.7)
Race, *n* (%)	
Black/African American	14 (16.1)
Native American	2 (2.3)
White	67 (77.0)
Other	4 (4.6)
Education, *n* (%)	
High school grad	21 (24.1)
Some college	23 (26.4)
Technical school/college grad	31 (35.6)
Master/advanced degree	12 (13.8)
HbA1c, % (SD)	8.8 (1.6)
BMI, kg/m^2^, *n* (SD)	34.8 (7.4)
Diabetes type, *n* (%)	
Type 1	10 (11.5)
Type 2	77 (88.5)
Diabetes duration, months (SD)	169.9 (145.2)
Diabetes care, *n* (%)	
Primary care	53 (60.9)
Diabetes specialist	34 (39.1)
Insulin therapy, *n* (%)	
Basal only	25 (28.7)^a^
MDI	62 (71.3)^b^
Daily SMBG, *n* (SD)	
Basal only	1.6 (0.6)
MDI	2.7 (1.8)

^a^ *n* = 25 T2D; ^b^*n* = 52 T2D.

BMI, body mass index; HbA1c, glycated hemoglobin; MDI, multiple daily insulin injection; SD, standard deviation; SMBG, self-monitoring of blood glucose; T2D, type 2 diabetes.

### Diabetes treatment satisfaction

**FIG. 2.**
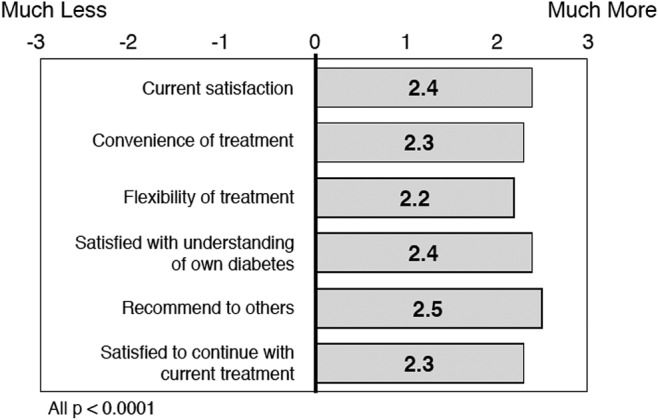
Diabetes Treatment Satisfaction Questionnaire item scores at month 6.

DTSQs results showed a high treatment satisfaction mean score at baseline: 29.8 ± 5.8 on a scale of 0–36 (0 = very dissatisfied, 36 = very satisfied). Significant improvements in treatment satisfaction (DTSQc) were observed at 6 months regardless of treatment modality (MDI or basal insulin only), diabetes type (T1D or T2D), or HCP practice type (specialist or nonspecialist), with a total mean (SD) score of 14.3 ± 5.1, <0.0001 (Fig. 2).

### Diabetes distress

Significant reductions in total mean (SD) DDS scores from baseline to 6 months were also observed: from 2.0 ± 0.8 to 1.7 ± 0.7, Δ −0.3, *P* < 0.0001. The reduction in regimen-related distress is notable: from “moderate distress” (2.4 ± 1.0) to “not distressed” (1.9 ± 0.9), Δ −0.5, *P* < 0.0001. Subgroup analysis showed significant reductions in distress regardless of treatment modality or HCP practice type; however, reduction in distress among T1D patients was not statistically significant (Table 2).

**Table 2.** Change in Diabetes Distress Scale Total Scores by Subgroup

*Subgroup*	n	*Baseline*	*6 months*	*Change*	P
MDI	58	2.1 (0.9)	1.7 (0.7)	−0.4 (0.6)	<0.0001
Basal only	22	1.8 (0.6)	1.5 (0.6)	−0.2 (0.4)	0.0414
T1D	9	1.9 (0.8)	1.8 (1.2)	0.0 (0.9)	0.9210
T2D	71	2.0 (0.8)	1.6 (0.6)	−0.4 (0.5)	<0.0001
Specialist HCP	32	1.8 (0.6)	1.6 (0.7)	−0.2 (0.6)	0.0444
Nonspecialist HCP	48	2.1 (0.9)	1.7 (0.7)	−0.4 (0.6)	<0.0001

HCP, healthcare professional's; T1D, type 1 diabetes.

### Glycemia

**FIG. 3.**
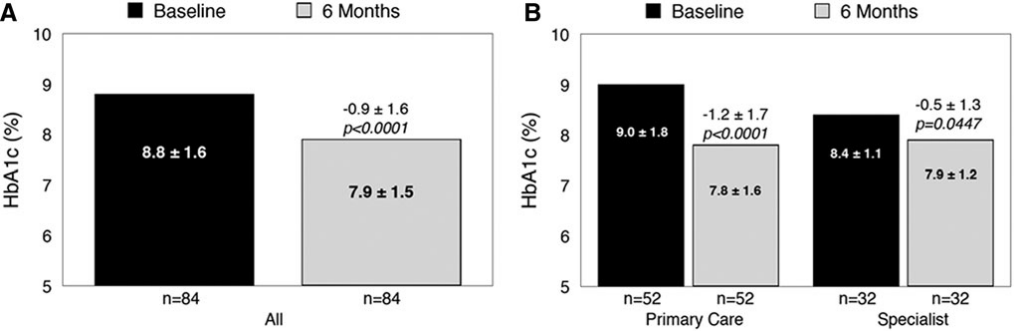
Change in mean HbA1c at month 6: overall **(A)** and by clinic setting **(B)**. HbA1c, glycated hemoglobin.

**FIG. 4.**
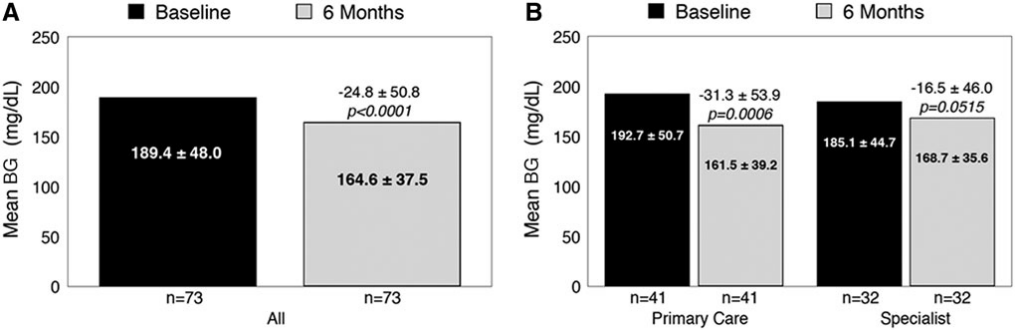
Change in mean BG at month 6: overall **(A)** and by clinic setting **(B)**.

Significant reductions from baseline in mean HbA1c (Fig. 3A) were observed within the entire cohort at month 6; however, the most notable improvements were seen among patients treated in primary care settings compared with diabetes specialty settings (Fig. 3B). Similar improvements were seen in mean BG (Fig. 4A), with the most notable improvements among primary care (nonspecialist) patients (Fig. 4B). Additional subanalyses showed no notable differences in HbA1c reductions between MDI- and basal-treated patients (−0.9% ± 1.5% vs. −1.0% ± 1.7%, respectively); however, T2D patients showed notably greater reductions than T1D patients (−1.0% ± 1.6% vs. −0.3% ± 1.0%). Among all patients, significant reductions in mean BG were observed at month 6: from 189.4 ± 48.0 to 164.6 ± 37.5 mg/dL, Δ 36.6 ± 12.9, *P* < 0.0001.

### SMBG frequency

Notable increases in daily SMBG frequency were observed between baseline (2.4 ± 1.7 checks per day) and month 3 (2.8 ± 2.1 Δ 0.4 ± 1.2, *P* = 0.0021) and month 6 (2.6 ± 2.0 Δ 0.2 ± 1.2, *P* = 0.1233).

### Sufficiency of diabetes data

An increase from baseline was observed in the number of patients with sufficient data at months 3 and 6 to make informed decision regarding treatment changes (Table 3). The increase was most notable among primary care clinicians from 22 at baseline to 50 patients (Δ 28) at month 3 and 52 patients (Δ 30) at month 6. There was no change among specialists. Sufficiency of data at and between clinic visits prompted an 84.2% increase in the number patients who received a medication change at months 3 and 6 compared to baseline.

**Table 3.** Number of Patients Who Received Treatment Interventions When Sufficient Data Were Available

	*Baseline,* n* = 87*	*Visit 3,* n* = 87*	*Visit 4,* n* = 87*
Sufficient data, *n* (%)	56 (64.4)	83 (95.4)	85 (97.7)
Any treatment change, *n* (%)	32 (57.1)	61 (73.5)	56 (65.9)
Adjusted diabetes medication	19 (33.9)	35 (42.2)	35 (41.2)
Addressed skill deficit	16 (28.6)	44 (53.0)	45 (52.9)
Addressed patient adherence	17 (30.4)	38 (45.8)	36 (42.4)
Counseled lifestyle changes	19 (33.9)	46 (55.4)	45 (52.9)

### Unscheduled medical visits/consults

A total of 130 unscheduled medical visits occurred during the study with 36 (41.1%) patients; 118 (90.8%) were initiated by clinicians and 107 (90.7%) of these were conducted remotely. Among the clinician-initiated remote visits, 103 (96.3%) were for medication changes and 3 (2.8%) involved education/training only. The 11 (9.3%) clinician-initiated in-clinic visits involved medication changes.

Notable differences in utilization of unscheduled visits within and between practice types were observed. Only one specialist made extensive use of unscheduled visits, mostly remote, compared with other specialists, whereas, most nonspecialist clinicians utilized unscheduled visits with their patients (Table 4).

**Table 4.** Unscheduled Medical Visits by Practice Type

				*Clinician-initiated USV*		
*HCP site*	*Enrolled patients (*n*)*	*Patients with ≥1 USV*	*Total USV (*n*)*	*All (*n*)*	*Clinic (*n*)*	*Remote (*n*)*
Specialist						
1	5	0	0	0	0	0
2	2	0	0	0	0	0
3	10	1	1	1	0	1
4	10	10	87	86	0	86
5	3	0	0	0	0	0
6	4	0	0	0	0	0
Subtotal	34	11	88	87	0	87
Nonspecialist						
1	9	8	13	6	1	11
2	1	0	0	0	0	0
3	7	5	13	11	0	13
4	22	8	9	9	7	2
5	14	4	7	5	3	3
Subtotal	53	25	42	31	11	20
Total	87	36	130	118	11	107

USV, unscheduled medical visits.

### Adverse events

There were no adverse device-related events (ADEs), serious ADEs, severe hypoglycemic episodes, or AEs leading to withdrawal nor fatal events reported during this investigation.

## Discussion

According to the most recent data, it is estimated that >50% of adults with diabetes are not achieving the HbA1c goal of <7.0%.^23,24^ A major contributor to poor glycemic control is difficulty adhering to prescribed self-management regimens. Suboptimal adherence is associated with long-term complications, more frequent hospitalizations, higher healthcare costs, and elevated mortality rates.^25–28^

Patient satisfaction with treatment and perceptions of the quality of their care is strongly associated with treatment adherence and clinical outcomes.^13,14,29,30^ Moreover, patients' understanding of the information they receive from their clinician correlates with both treatment satisfaction and communication.^31,32^

Several recent studies have demonstrated that timely and appropriate use of structured SMBG data improve glycemic control and reduce diabetes-related distress in both insulin-treated and noninsulin-treated diabetes.^1–4,33,34^ However, it is also well-documented that obtaining an accurate and complete BG data from traditional patient logbooks is problematic^5–7^ and inadequacy of data may contribute to clinician inertia in intensifying diabetes therapy.^8–12^

In this analysis of Accu-Chek Connect system use, we hypothesized that the availability of reliable, near real-time glucose data, transmitted automatically to clinicians in structured formats, may improve diabetes treatment satisfaction and improve clinical outcomes by prompting more frequent therapy adjustments and promoting more collaborative relationships between patients and their clinicians.

Our findings showed that use of the system was associated with significant improvements in treatment satisfaction, diabetes distress, and glycemic control. Moreover, the ability to more effectively monitor patient status through the web portal triage function facilitated remote diabetes management. As reported, of the 130 unscheduled medical visits observed, 118 were by clinicians and >90% of those visits were conducted remotely by over 50% of clinicians. This not only suggests that these clinicians were utilizing the “triage” function in the web portal home page to monitor patients but also indicates their perception of the high value and utility of remote consultations. This resulted not only in improved glycemic control but also reduced the burden of diabetes on patients as patients' clinic needs were met without the added time and inconvenience of coming to the clinic. It was somewhat surprising that only 33.3% patients in the specialty practice groups had an unscheduled medical visit, 10 of whom were treated by the same clinician, compared with 47.2% of primary care practice patients. This strongly supports the feasibility of system use in primary care settings.

Another surprising finding was the reduction in diabetes-related distress, specifically regimen distress. Because using a digital system required a certain “learning curve” for patients, we anticipated a slight increase in distress, which did not materialize.

In addition, although glycemic control was not a primary endpoint, it was gratifying to see improvements in this area, as well. This was particularly interesting because no additional training was provided to clinicians; we simply provided the tools that allowed them to more effectively monitor their patients' health status and then respond with appropriate therapy adjustments and counseling as needed.

As reported, the most notable improvements in glycemic control were seen among patients treated by primary care clinicians. Because the mean baseline HbA1c was notably higher in the primary care population compared with the specialty practice patients (9.0% ± 1.8% vs. 8.4% ± 1.1%), one would expect greater HbA1c reductions in the primary population. However, even with this difference, our study demonstrates that use of the system is both efficacious and feasible in busy primary care practices.

Some limitations are notable. The lack of a control group could be considered a limitation to our study; however, previous research has already demonstrated the clinical and psychosocial benefits of structured SMBG.^1–4,33,34^ Because our primary outcome was change in treatment satisfaction, we determined that a control group was not required for this study and a single arm study was sufficient, with each patient serving as his/her own control. Another limitation is our inability to directly measure reductions in acute glycemic events (hypoglycemia and hyperglycemia). Use of CGM in blinded mode would have allowed us to assess the impact of system use on reducing these events.

Despite these limitations, our analyses showed that use of the Accu-Chek Connect system is associated with increased treatment satisfaction, reduced distress, and improved glycemic control among individuals with insulin-treated diabetes. The Accu-Chek Connect system is a remotely connected digital tool that can contribute to positive clinical outcomes when the resulting data are used to inform appropriate treatment changes.
